# Flow Diversion for Reconstruction of Intradural Vertebral Artery Dissecting Aneurysms Causing Subarachnoid Hemorrhage—A Retrospective Study From Four Neurovascular Centers

**DOI:** 10.3389/fneur.2021.700164

**Published:** 2021-07-01

**Authors:** Jens Maybaum, Hans Henkes, Marta Aguilar-Pérez, Victoria Hellstern, Georg Alexander Gihr, Wolfgang Härtig, André Reisberg, Dirk Mucha, Marie-Sophie Schüngel, Richard Brill, Ulf Quäschling, Karl-Titus Hoffmann, Stefan Schob

**Affiliations:** ^1^Institute of Neuroradiology, University Hospital Leipzig, Leipzig, Germany; ^2^Neuroradiological Clinic, Katharinenhospital Stuttgart, Stuttgart, Germany; ^3^Paul Flechsig Institute for Brain Research, University of Leipzig, Leipzig, Germany; ^4^Department of Diagnostic Imaging and Interventional Radiology, Bergbau-Berufsgenossenschaft Hospital Bergmannstrost Halle, Halle, Germany; ^5^Department of Radiology, Interventional Radiology and Neuroradiology, Heinrich-Braun-Klinikum, Zwickau, Germany; ^6^Department of Neuroradiology, Radiology and Policlinic of Radiology, University Hospital Halle (Saale), Halle, Germany

**Keywords:** ruptured dissecting aneurysm, dominant vertebral artery dissection, endovascular reconstruction, subarachnoid hemorrhage, flow diverter

## Abstract

**Objective:** Dissecting aneurysms (DAs) of the vertebrobasilar territory manifesting with subarachnoid hemorrhage (SAH) are associated with significant morbi-mortality, especially in the case of re-hemorrhage. Sufficient reconstruction of the affected vessel is paramount, in particular, if a dominant vertebral artery (VA) is impacted. Reconstructive options include stent-assisted coiling and flow diversion (FD). The latter is technically less challenging and does not require catheterization of the fragile aneurysm. Our study aims to report a multicentric experience with FD for reconstruction of DA in acute SAH.

**Materials and Methods:** This retrospective study investigated 31 patients (age: 30–78 years, mean 55.5 years) who had suffered from SAH due to a DA of the dominant VA. The patients were treated between 2010 and 2020 in one of the following German neurovascular centers: University Hospital Leipzig, Katharinenhospital Stuttgart, BG Hospital Bergmannstrost Halle/Saale, and Heinrich-Braun-Klinikum Zwickau. Clinical history, imaging, implanted devices, and outcomes were reviewed for the study.

**Results:** Reconstruction with flow-diverting stents was performed in all cases. The p64 was implanted in 14 patients; one of them required an additional balloon-expandable stent to reconstruct severe stenosis in the target segment. One case demanded additional liquid embolization after procedural rupture, and in one case, p64 was combined with a PED. Further 13 patients were treated exclusively with the PED. The p48MW-HPC was used in two patients, one in combination with two additional Silk Vista Baby (SVB). Moreover, one patient was treated with a single SVB, one with a SILK+. Six patients died [Glasgow Outcome Scale (GOS) 1]. Causes of death were periprocedural re-hemorrhage, thrombotic occlusion of the main pulmonary artery, and delayed parenchymal hemorrhage. The remaining three patients died in the acute–subacute phase related to the severity of the initial hemorrhage and associated comorbidities. One patient became apallic (GOS 2), whereas two patients had severe disability (GOS 3) and four had moderate disability (GOS 4). Eighteen patients showed a complete recovery (GOS 5).

**Conclusion:** Reconstruction of VA-DA in acute SAH with flow-diverting stents is a promising approach. However, the severity of the condition is reflected by high overall morbi-mortality, even despite technically successful endovascular treatment.

## Introduction

Intracranial dissections of the vertebral artery (VA) represent rare but potentially critical cerebrovascular lesions associated with a significant variety of unspecific symptoms (1). The dissection of an intracranial VA may remain clinically silent but more frequently manifests with posterior circulation stroke, subarachnoid hemorrhage (SAH), or, less frequently, spinal ischemia (2, 3). More than 80% of patients with intracranial VA dissections of the steno-occlusive type develop posterior circulation stroke. However, the majority of those improve without the imperative for endovascular treatment (4, 5).

Ruptured dissecting aneurysms of the intracranial VA are associated with worse outcomes. Between 24 and 72 h after the segmental vascular injury, frequently indicated by a characteristic occipital and nuchal headache, severe SAH manifests in almost every case (6). Subsequently, re-hemorrhage occurs in more than 70% of patients, culminating in mortality rates of ~50% (7). As a consequence, early and sufficient therapy of ruptured dissecting aneurysms of the intracranial VA is mandatory.

Depending on the hemodynamic situation in the posterior circulation and the localization of the ruptured dissecting aneurysm, different endovascular approaches must be considered (8, 9). In case the rupture site is associated with a hypoplastic VA, segmental sacrifice, ideally sparing the posterior inferior cerebellar artery (PICA) orifice, has shown promising results (8, 10). However, segmental sacrifice and proximal VA occlusion carry significant risk for ischemia and, in some cases, re-bleeding (11).

In particular, if the ruptured dissecting aneurysm arises from a dominant VA or involves the PICA origin, a reconstructive technique is recommendable (11, 12). Reconstruction can be achieved with different approaches, for example, stent-in-stent implantation, stent-assisted coiling, and flow-diverting stents (12–16). However, related to the rarity of the condition, only retrospective reports on the different strategies exist, and the most suitable treatment remains to be determined (17).

Flow-diverting stents offer several advantages over the alternative endovascular techniques; most importantly, they allow the reconstruction of the vessel without primary catheterization of the highly fragile dissecting aneurysm, and their increased surface coverage provides a superior seal of the potentially extensive intimal tear in comparison to conventional, low-porosity laser-cut stents. However, reports on flow diversion (FD) in this specific context are lacking.

This study, therefore, aims to report our multicenter experience of FD for the reconstruction of acutely ruptured, dissecting aneurysms of the dominant intracranial VA, including clinical and procedural aspects as well as follow-up data in order to present feasibility, safety, and effectiveness of this approach.

## Materials and Methods

Our retrospective study of multicenter data regarding the reconstructive approach with flow-diverting stents to treat ruptured dissecting aneurysms of the vertebrobasilar system was approved by the institutional ethics committee (local institutional review board, IRB, nr. AZ 208-15-0010062015). The patients were treated between 2010 and 2020 in one of the following German neurovascular centers: University Hospital Leipzig (*n* = 13), Katharinenhospital Stuttgart (*n* = 16), BG Klinikum Bergmannstrost Halle/Saale (*n* = 1), and Heinrich-Braun-Krankenhaus Zwickau (*n* = 1). Informed consent of each patient regarding the use of radiological and clinical data was obtained in written form by either the patient or his or her legal representative.

Clinical, procedural, and imaging data including anatomical aspects of the aneurysm (size, location, and morphology), post-procedural aneurysmal status, devices used, technical aspects, and clinical follow-up data using the modified Rankin scale (mRS) were analyzed. Any clinical events in the postoperative course were documented. Initial and follow-up occlusion rates were graded according to the O'Kelly-Marotta (OKM) grading scale, as reported previously (18).

Platelet function testing was not mandatory and was routinely performed only in one center (Katharinenhospital Stuttgart, 9/16 patients). No cases of hypo-response were recorded in the included patients. Dual platelet inhibition was performed in all patients if necessary. Those patients who received platelet function testing and revealed no insufficient response were treated with a combination of Clopidogrel (1 × 75 mg PO daily) and acetylsalicylic acid (ASA) (1 × 100 mg PO daily). The remaining 15 patients received a combination of Ticagrelor (2 × 90 mg PO, bid) and ASA (1 × 100 mg PO daily). Ticagrelor was chosen as a simple measure to avoid insufficient platelet function inhibition, in line with earlier studies (19).

All interventions were performed under general anesthesia using biplane neuroangiography suites. In 19 cases, a triaxial system with guiding catheter, distal access catheter (11× 6F SOFIA, MicroVention, Alajuela, Costa Rica; 8× 6F Heartrail II, Terumo Europe, Belgium), and microcatheter was used. In an additional 12 cases, a coaxial setup consisting only of guiding catheter and microcatheter was applied. Guiding catheters used were 13× 6F Neuron Max (Penumbra, Alameda, USA), 7× 6F Guider Softtip (Boston Scientific, Marlborough, USA), and 11× 6F Envoy MP (Cerenovus, Irvine, CA USA).

As each flow diverter has its specific requirements for delivery, the microcatheters were chosen accordingly. The Pipeline Embolization Device (Medtronic, Irvine, USA) was implanted using the Phenom™ 27 (Medtronic, Irvine, USA) microcatheter. The p64 Flow Modulation Device (phenox, Bochum, Germany) was implanted using the Excelsior XT 27 (Stryker Neurovascular, Fremont, USA) microcatheter, whereas the novel p64MW-HPC was implanted using the Rebar 18 (Medtronic, Irvine, USA). The Silk+ (Balt Extrusion, Montmorency, France) was implanted using the Vasco 25 (Balt Extrusion, Montmorency France) microcatheter. The p48MW (phenox, Bochum, Germany) was implanted using the Prowler Select Plus (Cerenovus, Irvine, CA, USA) microcatheter. The Silk Vista Baby (SVB) (Balt Extrusion, Montmorency, France) was implanted *via* a Headway 17 (MicroVention, Tustin, USA) microcatheter.

Table 1 summarizes the relevant information of all included patients.

**Table 1:** Clinical data of all included patients.

**Case**	**Sex**	**Age**	**Location, hemodynamic situation**	**Hunt and Hess**	**Fisher grade**	**Lesion dimension (mm)**	**Pseudo aneurysm max. diameter (mm)**	**Endovascular approach**	**EVD**	**Craniectomy**	**GOS at time of review**
1	Male	30	Dominant left vertebral artery; right hypoplastic	IV	4	14 × 2	9	Flow Diverter + Coiling (1 × p64)	Right frontal, VP-Shunt	No	4
2	Male	57	Dominant right vertebral artery; left hypoplastic	IV	4	11 × 2	4	Flow Diverter (2 × PED)	Right frontal	No	4
3	Male	48	Dominant right vertebral artery; codominant left	III	4	10.7 × 3	4.7	Flow Diverter (4 × p64)	Right frontal	No	5
4	Male	78	Dominant left vertebral artery; right hypoplastic V4	V	4	30 × 4	8	Flow Diverter + Drug Eluting Stent (9 × p64, 1 × Corofle × ISAR)	Right frontal	No	1
5	Male	40	Dominant right vertebral artery; codominant left	III	4	9 × 3	3	Flow Diverter (1 × PED)	None	No	5
6	Male	52	Left dominant vertebral artery; codominant right	IV	3	7 × 2	1.3	Flow Diverter (1 × p64)	Left frontal	No	5
7	Male	67	Left dominant vertebral artery; codominant right	IV	4	14 × 2	3	Flow Diverter (1 × p64)	Bifrontal, VP-Shunt	No	5
8	Male	78	Hypoplastic right vertebral artery with PICA ending; dominant left vertebral artery	1	2	20 × 4	8	Flow Diverter + Coiling (2 × p64)	None	No	1
9	Female	57	Dominant left vertebral artery; codominant right	III	4	19 × 2	4	Flow Diverter (1 × p64, 1 × PED)	Right frontal, VP-Shunt	No	4
10	Female	66	Dominant right vertebral artery; left hypoplastic V4	III	4	28 × 4	6	Flow Diverter + Liquid Embolizate (2 × p64)	None	No	1
11	Male	58	Dominant right vertebral artery; codominant left	I	2	18 × 4	4	Flow Diverter (2 × PED)	None	No	5
12	Male	51	Dominant left vertebral artery; codominant right	V	4	24 × 3.5	6	Flow Diverter (2 × PED)	None	No	5
13	Male	49	Dominant right vertebral artery; hypoplastic left vertebral artery with PICA ending	IV	4	18 × 3	7	Flow Diverter (1 × PED)	Left frontal	No	5
14	Male	67	Dominant left vertebral artery; codominant right	II	3	15 × 2	2	Flow Diverter (1 × p64)	None	No	3
15	Male	57	Dominant right vertebral artery; dissection stenosis vertebral artery left	I	2	18 × 4	6	Flow Diverter (1 × p64)	None	No	5
16	Female	41	Dominant left V4 with equally important right V4	III	4	12 × 4		Flow Diverter (2 × p64)	None	No	5
17	Female	54	Dominant right vertebral artery; codominant left	I	4	8 × 3	4,5	Flow Diverter (1 × p64)	None	No	5
18	Male	53	Dominant left vertebral artery; right hypoplastic vertebral artery	II	4	12 × 4	4	Flow Diverter (2 × p64)	VP-Shunt	No	5
19	Female	67	Dominant left vertebral artery; codominant right	IV	4	10 × 3	5	Flow Diverter + Coiling (1 × PED)	Right frontal, VP-Shunt	Yes	5
20	Female	52	Dominant left vertebral artery; hypoplastic right vertebral artery with PICA ending	IV	4	12 × 4	6	Flow Diverter (3 × PED)	Right frontal, VP-Shunt	No	1
21	Male	44	Dominant left vertebral artery; codominant right	IV	4	19 × 3	2	Flow Diverter (2 × PED)	None	No	5
22	Male	50	Dominant left vertebral artery; codominant right	I	4	9 × 3	2.3	Flow Diverter (1 × PED)	None	No	5
23	Male	67	Dominant left vertebral artery; codominant right	III	4	21 × 3	3	Flow Diverter (2 × PED)	Right frontal, VP-Shunt	No	5
24	Female	66	Dominant left vertebral artery; codominant right	IV	4	8 × 4	4	Flow Diverter (3 × PED)	None	No	
25	Female	71	Dominant left vertebral artery; codominant right	III	4	30 × 4	3	Flow Diverter (5 × PED)	Left frontal	No	1
26	Female	57	Dominant right vertebral artery; left hypoplastic	No relation to dissection		17 × 4	4	Flow Diverter (1 × PED)	None	Yes	3
27	Female	47	Dominant right vertebral artery; left hypoplastic	IV	3	42 × IV	6.6	Flow Diverter + Coronary stent (8 × p48MW_HPC, 2 × SVB, 1 × Rebel)	Left frontal	Yes	1
28	Female	44	Dominant left vertebral artery; right hypoplastic V4	–	–	30 × 4	4	Flow Diverter (1 × p48MW_HPC)	None	No	2
29	Female	69	Dominant left vertebral artery; right hypoplastic	–	–	11.5 × 4	7	Flow Diverter (1 × Silk)	None	No	5
30	Female	35	Dominant right vertebral artery; equally strong left	II	4	15 × 3	3	Flow Diverter (1 × SVB)	None	No	5
31	Female	49	Dominant left vertebral artery; hypoplastic right	III	4	11.5 × 4	4	Flow Diverter (1 × p64)	None	No	5

## Results

### Patients

Thirty-one patients (17 male and 14 female) between 30 and 78 years who had suffered from SAH caused by the rupture of a dissecting aneurysm of the dominant intradural VA were included. Of those, 11 had the dissecting aneurysm at the right-hand side dominant VA, while the remaining patients had the dissecting aneurysm at the left-hand side dominant VA. In six patients, the dissecting aneurysm morphologically involved the basilar artery.

### Implanted Devices and Adjunctive Techniques

**Figure 1:**
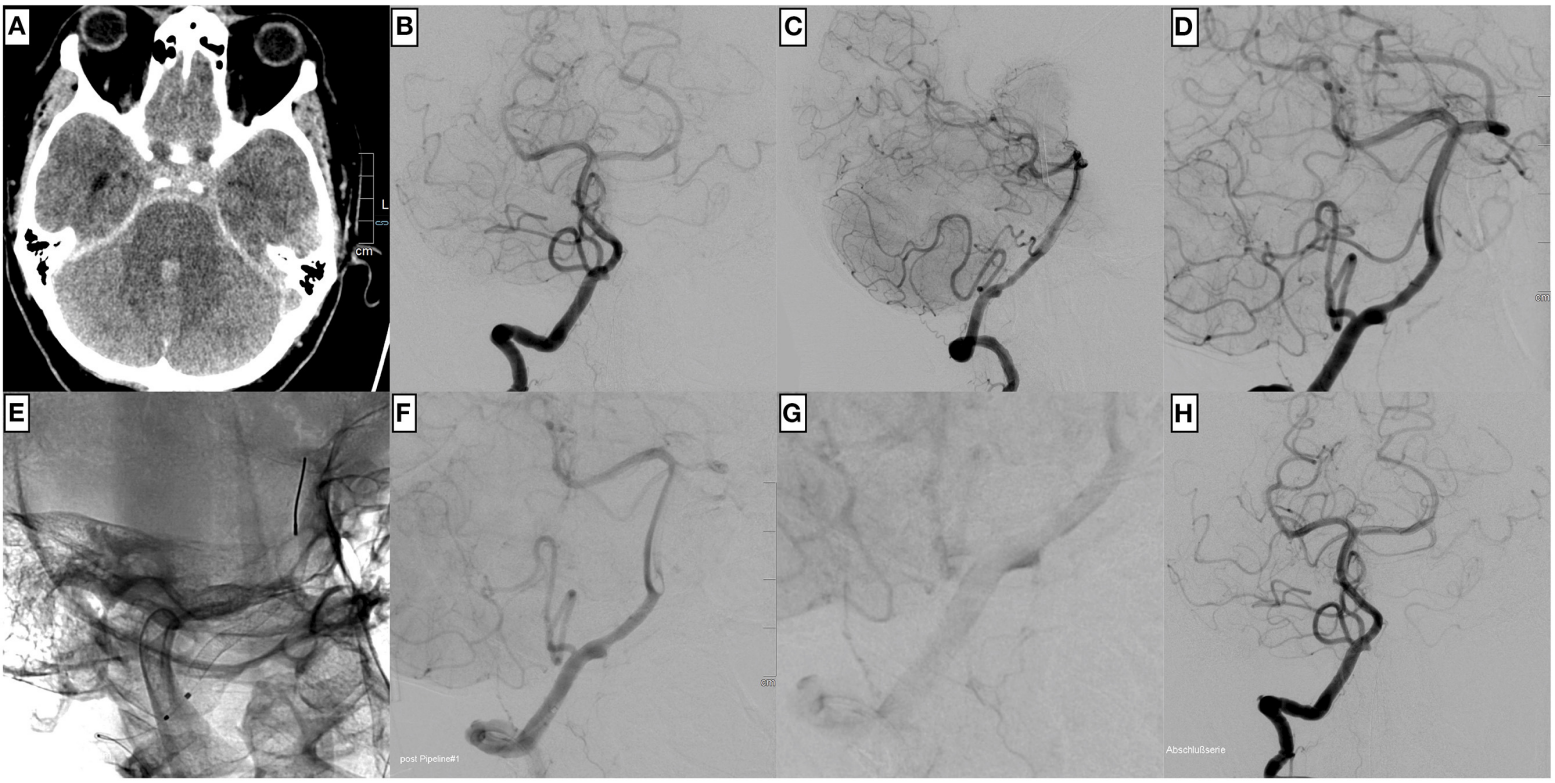
An example of uncomplicated PED implantation for treating a ruptured, dissecting aneurysm of the right dominant intradural vertebral artery in a 57-year-old male patient. **(A)** Non-enhanced cranial computed tomography shows Fisher grade 3 subarachnoid hemorrhage. **(B)** The injection of the right vertebral artery in posterior–anterior, **(C)** lateral, and **(D)** working projection demonstrates the comparatively confined ruptured dissecting aneurysm close to the posterior inferior cerebellar artery (PICA) orifice. After unimpeded catheterization with a Phenom™ 27, a PED **(E)** is implanted. The flow diverter is centered over the dissecting aneurysm. The control injection **(F)** shows the reconstruction of the vessel, now without irregularities in the post-PICA segment, which were apparent before implantation **(D)**, and significant stasis of contrast agent within the aneurysm **(G)**. The final angiogram in posterior–anterior projection **(H)** reveals timely opacification of the posterior circulation.

**Figure 2:**
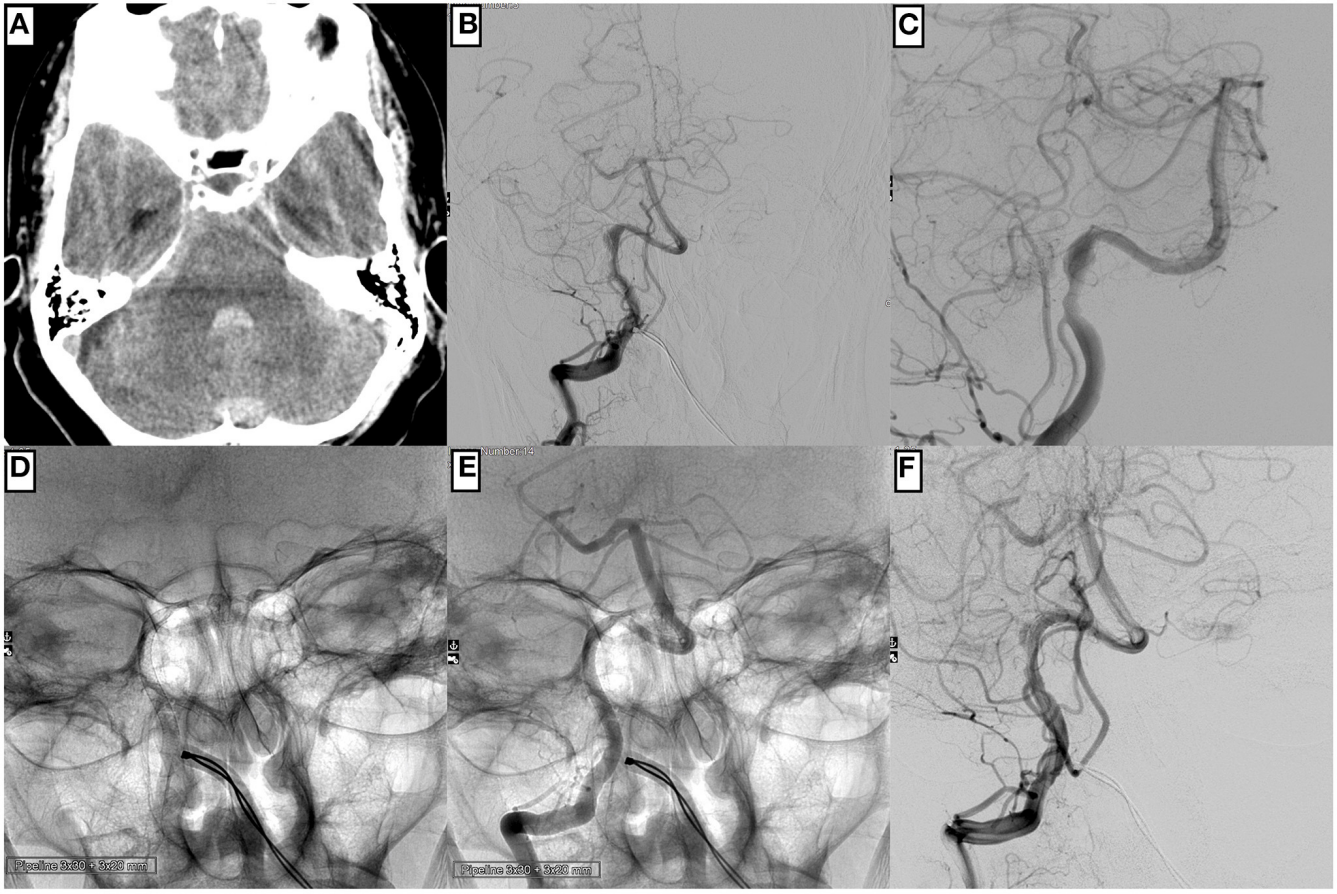
An example of technically unremarkable PED implantation is to reconstruct a right-hand side distal V4 segment affected by an extensive dissecting aneurysm in a 58-year-old male patient. **(A)** Cranial computed tomography prior intervention displays SAH Fisher grade 3. The injection of the right-hand side vertebral artery in posterior–anterior **(B)** and working projection **(C)** demonstrates an extensive, hourglass-shaped dissecting aneurysm directly distal to the PICA orifice. After uneventful catheterization with a Phenom™ 27 microcatheter, two PED flow diverters are implanted in telescoping fashion **(D,E)**. The control injection demonstrates timely opacification of the PICA and the distal vertebrobasilar vessels together with delayed and prolonged opacification of the pseudoaneurysm **(F)**.

Reconstruction with one flow-diverting stent was sufficient in 15 cases. A single p64 was used in seven patients and one PED was used in five patients, whereas a single p48 and a single SVB were applied in one case each. Reconstruction with two flow diverter stents in overlapping fashion was necessary for 10 patients. Of those, 2× p64 in overlapping fashion were used in four patients, 2× PED in overlapping fashion was implanted in five further patients, and 1× p64 together with 1× PED were implanted in one patient. Examples of endovascular reconstruction with overlapping flow diverters of a relatively confined and extensive dissecting aneurysm are given in Figures 1, 2. Multiple overlapping flow diverter stents were implanted in the remaining six patients. One patient was treated with five overlapping PED flow diverters, and two patients were treated with three overlapping PED flow diverters. One additional patient received four overlapping p64 flow diverters, and the next patient required nine overlapping p64 flow diverters together with a balloon-mounted coronary stent. A further patient was treated with eight overlapping p48MW-HPC combined with two additional SVB flow diverters and one balloon-mounted coronary stent. In the last two patients, the dissecting aneurysm had associated high-grade stenosis, which required implantation of a balloon-mounted coronary stent to prevent occlusion of the respective segment. Balloon angioplasty was necessary in six other cases (4× p64, 1× p48MW, and 1× PED) to achieve sufficient wall apposition of the implanted flow diverters after initially insufficient opening.

**Figure 3:**
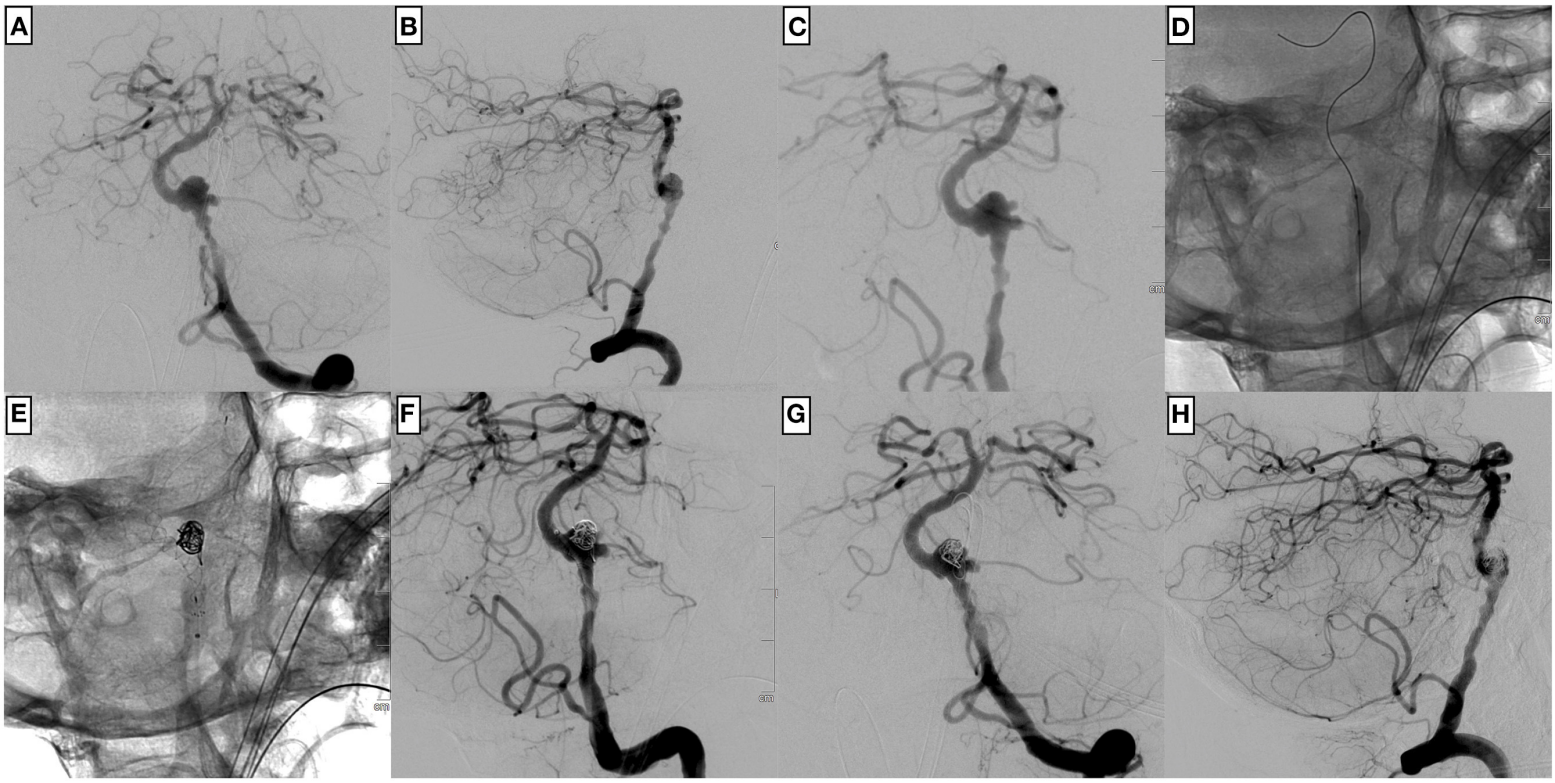
An example of a complex endovascular treatment including balloon angioplasty, coiling in jailing technique, and p64 implantation of a large ruptured, dissecting aneurysm of the left dominant intradural vertebral artery in a 78-year-old male patient. **(A)** The injection of the left vertebral artery in posterior–anterior, **(B)** lateral, and **(C)** working projection demonstrates an extensive, multi-lobulated dissecting aneurysm close to the vertebral artery junction. A stenosis proximal to the aneurysm requires balloon angioplasty **(D)** before implanting the flow diverter. **(E)** Few coils are placed in jailing position within the large pseudoaneurysm to promote thrombus formation and reduce the risk for re-rupture. The p64 was distally anchored within the basilar artery, and the proximal landing zone was defined slightly above the PICA orifice. The control injection **(F–H)** shows a less irregular shape of the affected V4 segment and a still markedly opacified pseudoaneurysm.

**Figure 4:**
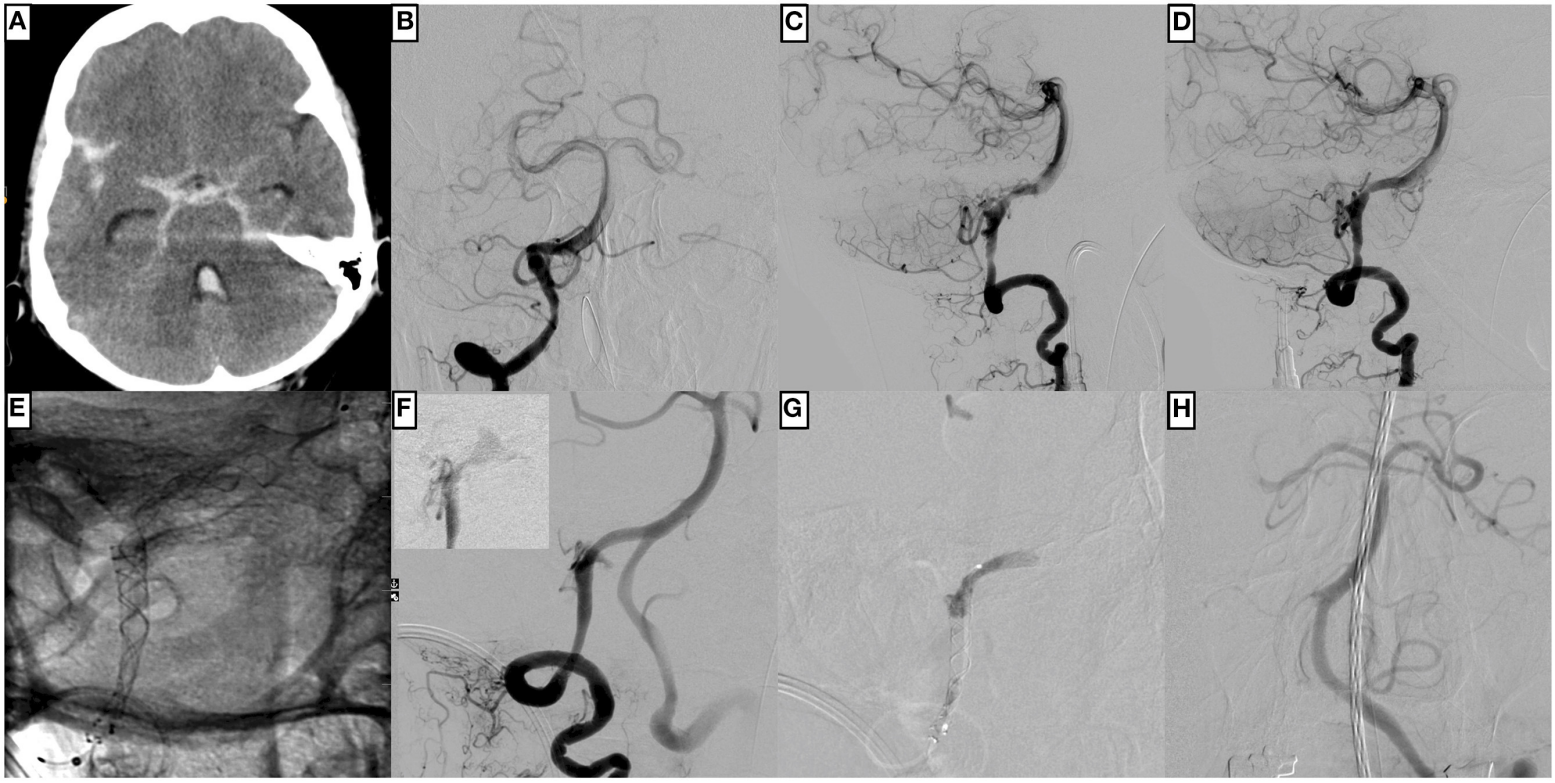
An example of complicated p64 implantation for treating a ruptured, dissecting aneurysm of the right dominant intradural vertebral artery in a 66-year-old male patient. **(A)** Non-enhanced cranial computed tomography shows Fisher grade 4 subarachnoid hemorrhage. **(B)** The injection of the right vertebral artery in the posterior–anterior and **(C)** lateral projection demonstrates the underlying fusiform dissecting aneurysm with the PICA at its center. **(D)** represents the working projection for flow diverter implantation, which is subsequently evaluated in **(E)**; two p64 are implanted in telescoping technique with sufficient overlap of both devices at the center of the dissecting aneurysm. Note the distal intraluminal position of the p64 wire and the olive at its tip. **(F)** shows the control injection moments after unremarkable p64 implantation, revealing significant contrast extravasation from the distal V4 segment (the smaller image in the upper left corner shows extravascular pooling of contrast agent seconds later). The procedural re-rupture after flow diverter implantation prompted embolization of the respective segment with Histoacryl, which immediately stopped the bleeding **(G)**. A small proportion of the liquid embolic agent dislocated into the right posterior cerebral artery. The control injection *via* the left vertebral artery **(H)** demonstrates the basilar artery's timely perfusion and branches, except for the right posterior cerebral artery, which exhibits a slightly delayed filling.

Additional occlusive techniques—coiling and liquid embolization—were necessary in four cases. Coiling was performed based on the jailing technique in three patients, aiming for enhanced thrombosis of the large pseudoaneurysm in all of those cases. An exemplary case is shown in Figure 3. Liquid embolization resulting from periprocedural re-rupture was necessary in one case, which is demonstrated in Figure 4.

### Ischemic Complications

In this series, 9.6% (3/31) of the patients experienced an ischemic stroke. Two of them suffered from a partial PICA infarction, and one developed a subtle thalamic stroke. One of the PICA infarctions occurred in a patient with a fusiform dissecting, partially thrombosed aneurysm extending into the basilar artery, responsible for a Hunt and Hess grade V SAH. The patient succumbed to the severity of the hemorrhage in the early post-interventional phase (11 days after treatment), and the PICA infarct was irrelevant to the outcome. The two remaining patients had good outcomes. One of both developed a partial PICA infarct due to an in-stent thrombosis within a PED2 shield 3 days after the implantation. Platelet function inhibition had been initiated with acetylsalicylic acid (ASA, 500 mg IV per day) only in order to reduce the risk for hemorrhage, as was suggested earlier (20). The in-stent thrombosis was treated successfully with IV application of eptifibatide according to the manufacturer's instruction. The patient experienced an overall good recovery [Glasgow Outcome Scale (GOS) 4].

The third patient suffered from thalamic infarction secondary to post-hemorrhagic vasospasm in the vertebrobasilar territory, from which he recovered utterly (GOS 5).

### Hemorrhagic Complications and Re-hemorrhage

Hemorrhagic complications, on the other hand, occurred in three further patients. One patient suffered from a periprocedural re-rupture of the dissecting aneurysm. Salvage embolization with n-butyl cyanoacrylate (Histoacryl, B. Braun) was performed immediately, but the patient succumbed to the sudden rise of intracranial pressure. The second patient suffered from cerebellar hemorrhage within the first 24 h after flow diverter implantation and did not recover well from the hemorrhage (GOS 2). The third patient experienced a large parenchymal hemorrhage 3.5 months after the endovascular therapy while being under dual antiplatelet therapy (ASA and Clopidogrel, dosage according to the manufacturer's instruction) and died in the aftermath of this event (GOS 1).

### Outcomes

Six patients died (GOS 1), two of those patients in the context of hemorrhagic complications. Thereby, one case was related to a periprocedural re-hemorrhage, which was angiographically controlled with immediate liquid embolization after flow diverter implantation but culminated in uncontrollable high intracranial pressure, as demonstrated in Figure 4. The second case suffered from delayed major parenchymal hemorrhage 3.5 months after successful endovascular treatment. A third patient, Hunt and Hess SAH grade I, developed a fulminant and eventually fatal pulmonary embolism. The fourth patient, who had suffered from a sizeable dissecting aneurysm extending from the V3 segment into the basilar artery, died within the first week after reconstruction due to repeated episodes of uncontrollable intracranial pressure. The fifth patient had suffered from SAH Hunt and Hess grade IV and depended on a left-ventricular assist device, and therefore required dual antiplatelet medication together with oral anticoagulation, and died after discharge from the hospital without, in retrospect, precisely determinable cause. The last patient of the GOS 1 group presented with Hunt and Hess grade V and developed an outcome-wise insignificant PICA infarction after treatment before he succumbed to the severity of the SAH. The only GOS 2 case resulted from early re-hemorrhage within 24 h after treatment. Two patients had severe disabilities (GOS 3), in one case as a result of the initial ictus, whereas the other patient already presented with a reduced general condition (alcoholism) and required permanent ventriculoperitoneal shunting.

The remaining three patients died in the acute–subacute phase related to the severity of the initial hemorrhage and associated comorbidities. One patient became apallic (GOS 2) as a consequence of re-hemorrhage within 24 h post-procedure. Two patients had severe disability (GOS 3) and four had moderate disability (GOS 4). Eighteen patients (58.1%) showed a complete recovery (GOS 5).

## Discussion

This study summarizes our multicenter experience with flow diverter implantation, using different flow diverter models, to treat acutely ruptured dissecting aneurysms of dominant intracranial vertebral arteries.

Flow-diverting stents are designed to reconstruct parent vessels of cerebral aneurysms. The reconstruction after implantation is achieved stepwise. Firstly, the dense mesh of the flow diverter covering the aneurysmal entry reduces inflow and causes redirection of blood flow along the physiological axis of the parent vessel (21). That way, intra-aneurysmal pressure, and thus transmural force, is reduced immediately. Subsequently, a thrombus is formed in the aneurysm sac, and neointima formation along the lattice of the flow diverter begins (22). This concept has been proven to be clinically successful for treating anatomically challenging aneurysms in the anterior circulation, particularly for wide-neck sidewall or complex fusiform aneurysms, and long-term follow-up data after flow diverter implantation substantiate good safety and efficacy (23, 24). However, ruptured dissecting aneurysms of the intradural VA are biologically distinct from incidental aneurysms, particularly the anterior circulation, and very little data on the use of flow diverters for treatment of dissecting intracranial VA aneurysms have been made available (16). Considering the data mentioned above, together with the imperative for immediate reconstruction of dominant V4 segments affected by ruptured dissecting aneurysms, further investigations of FD for ruptured dissecting VA aneurysms are required (8).

Overall, the results of our study underline the safety and feasibility of FD as a strategy for the treatment of acutely ruptured dissecting aneurysms affecting dominant and thus indispensable vertebral arteries. However, significant clinical adverse events, as well as technical adverse events, must be reflected critically. Therefore, those points are addressed in the following paragraphs.

Morbidity and mortality associated with ruptured dissecting aneurysms of the VA are mainly related to the severity of the initial hemorrhage, ischemic complications, the occurrence and magnitude of re-hemorrhage, and comorbidities (6, 25).

### Ischemic Complications

In our cohort, <10% of the patients suffered from posterior circulation ischemic stroke in association with the SAH, its treatment, or its early sequelae. Two of the infarctions affected the PICA territory, but neither those nor the singular thalamic infarction were relevant for the individual outcome. Recent reports indicated that the major branches of the intradural VA, most notably the PICA, remain patent and functionally unaffected in most cases after flow diverter implantation (26, 27). Our results are not entirely in accordance with those reports, underlining the pathophysiological inequality of ruptured dissecting aneurysms of the intracranial VA compared to electively treated aneurysms in the exact location. More specifically, the different fate of the PICA in our patients is explainable as follows. Firstly, it is comprehensible that the dissection itself or the mass effect of the dissecting aneurysm can affect the PICA directly and therefore cause significant stenosis or even occlusion with subsequent infarction. Secondly, acute SAH is associated with significant and prolonged platelet activation and aggregation, facilitating device-associated thrombo-embolism and occlusion of covered side branches (28). Aside from that, our findings suggest that single antiplatelet therapy, instead of dual antiplatelet therapy, after implantation of devices with reduced thrombogenicity due to hydrophilic coating, must be evaluated critically in acute SAH. However, several investigations suggested the feasibility and safety of this approach (25, 29). Prior experience has shown that the hemorrhage-induced platelet activation requires a tailored dosage based on response tests (e.g., Multiplate, Roche Diagnostics; VerifyNow, Accriva), with prasugrel being more efficient than ASA (30).

### Hemorrhagic Complications

Significant hemorrhagic complications occurred in the same proportion as ischemic complications, affecting 3/31 patients. However, contrary to the ischemic complications, all hemorrhagic complications were causative for the death of the respective patient or persisting and severe neurological deficit. Our findings in this regard are in line with earlier reports, underlining the significance of re-hemorrhage for the patient's outcome (31). Concerning the time point of hemorrhagic complications, our results are also in accordance with earlier studies showing that hemorrhagic complications occur either during intervention or in the vulnerable early phase post-intervention (17, 32, 33). However, few reports also showed markedly delayed parenchymal hemorrhages with insignificant distance to the dissecting aneurysm—which we encountered in one patient after the implantation of a PED2 shield together with additional coiling. They hypothesized an association with hemorrhagic transformation of small, imaging-negative lesions under dual antiplatelet therapy (34).

### Outcome—Synopsis

Overall, 9/31 patients (29%) had unfavorable outcomes (GOS 1–3). Five of the six patients who died (GOS 1) in the context of the SAH already presented with severe deficits (Hunt and Hess grade III-V). Five of them also required treatment with adjunctive techniques (1× Histoacryl embolization, 2× coiling, and 2× coronary stent to reconstruct high-grade stenosis of the flow diverter-bearing segment), indicating technically particularly complicated cases. One of the six GOS 1 patients presented with Hunt and Hess grade I SAH but developed a fulminant and eventually fatal pulmonary embolism, an infrequent but recognized complication in acute SAH (35). The only GOS 2 case resulted from early re-hemorrhage within 24 h after treatment. The remaining 22 patients either recovered completely (*n* = 18) or regained independence in their daily routine (GOS 4: *n* = 4). In summary, high Hunt and Hess grades and the necessity for adjunctive techniques in ruptured dissecting VA aneurysms and respective comorbidities were associated with poor outcomes in our patients, according to earlier studies (11, 17, 30).

### Technical Issues and Device Considerations

A total of six cases required additional endovascular maneuvers related to purely technical issues. In two patients, foreshortening of the flow diverter (1× PED2; 1× p64) resulted in insufficient coverage of the proximal portion of the dissecting aneurysms and required implantation of a second device in telescoping technique. In two other cases, the insufficient opening of the device (1× p64: distal landing zone; 1× PED 2: mid-section) demanded balloon angioplasty to achieve adequate wall apposition. These four procedures were not associated with unfavorable outcomes. In the remaining two cases, the dissecting aneurysm exhibited a partly stenotic portion that compressed the flow diverter construct and required implantation of a balloon-expandable coronary stent to prevent impending occlusion. Despite the technical success, both patients died within the first week post-procedure.

Notably, all flow-diverting stents applied in the present study were sufficiently implantable and technically effective for treating the respective dissecting aneurysm. However, depending on the anatomy of the target vessel, the hemodynamic situation at hand, together with the necessity for subsequent surgery and relevant comorbidities, device selection can be decisive for the outcome. Therefore, the subsequent consideration aims to summarize our experience with the different device models in acutely ruptured dissecting VA aneurysms.

FD in acute SAH requires dual antiplatelet therapy, which delays obliteration of the aneurysm, increases the risk for re-rupture, and has been associated with a complication rate of 18% (36). In this regard, the selection of a coated device with biomimicry properties that prevent interactions with blood cells—most importantly platelets—is advantageous, as this strategy allows early reduction of platelet function inhibition or even single antiplatelet treatment (25). Current options are the Pipeline Flex with Shield Technology, the p48MW HPC, and the p64MW HPC. In our experience, the PED shield practically displays the most significant empirical outward force with optimal wall apposition—and thus the reconstructive potential for intradural dissecting aneurysms. However, the device requires a 0.027” inner diameter (ID) microcatheter for implantation, which is comparatively stiff, less maneuverable than a 0.021” microcatheter, and can therefore be distinctly problematic in challenging segments, especially in a situation with a preexisting mural injury.

The p48MW HPC and the p64MW HPC only require a 0.021” ID, and therefore a more versatile microcatheter, and come with a movable inner wire that can be placed up to 6 cm distally within the target vessel. This setup not only stabilizes the system during implantation (37), it also can be used to navigate the microcatheter through and distal to the implanted stent after implantation without losing access to the true lumen, which is a beneficial feature in large-scale dissecting aneurysms (21). However, in our experience, balloon angioplasty is required more frequently to achieve sufficient wall apposition when implanting a p48MW HPC or a p64MW HPC compared to a PED Flex Shield.

Although not available with anti-thrombotic surface modification, the SVB can be the device of choice for dissecting aneurysms of the intracranial VA and basilar artery. The low-profile flow diverter is designed for the treatment of vessels ranging from 1.5 to 3.5 mm diameter and can be implanted *via* a 0.017” microcatheter. This feature is uniquely advantageous in challenging anatomies, as the 0.017” microcatheter allows atraumatic navigation in very elongated and curved vessels and provides a maximum of controllability (38). Remarkably, the SVB also achieves high rates of early obliteration in challenging cerebral aneurysms (38).

The Silk+, representing an older-generation flow-diverting stent, requires a rather stiff 0.025” microcatheter, which can be a significant limitation in the posterior circulation anatomy and especially in case of VA-DA. However, if a VA with a large diameter requires FD, the Silk+ may be the device of choice, as it is available in dimensions up to 5.5 × 40 mm and can be used to treat segments of 5.75 mm diameter.

## Conclusion

Reconstructive treatment in ruptured dissecting VA aneurysms of the dominant VA with flow-diverting stents is a technically safe and effective approach; however, the severity of the condition is reflected by high rates of morbidity and mortality, even despite technically successful endovascular treatment. Different flow-diverting stents are available, and case-adapted device selection is essential, as each flow diverter has a unique combination of features. In our experience, the size of the microcatheter required for implantation, anti-thrombotic surface modification, and radial force are the most significant features that should be taken into consideration when choosing the flow diverter model for treatment of ruptured dissecting VA aneurysms.

## Data Availability Statement

The original contributions presented in the study are included in the article/supplementary material, further inquiries can be directed to the corresponding author/s.

## Ethics Statement

The studies involving human participants were reviewed and approved by Ethics Committee of University Hospital Leipzig. The patients/participants provided their written informed consent to participate in this study.

## Author Contributions

JM, HH, WH, K-TH, and SS designed the manuscript and drafted the final version. HH, UQ, SS, JM, VH, MA-P, DM, AR, and K-TH performed the interventions. M-SS, GG, JM, and RB were responsible for data curation and statistical analysis. M-SS and SS designed the figures. All authors contributed to the article and approved the submitted version.

## Conflict of Interest

MA-P has proctoring and consultancy agreements with Kaneka, Medtronic and phenox. VH has consultancy agreements with phenox. HH is co-founder and shareholder of phenox. UQ has proctoring and consultancy agreements with phenox and Balt Germany. SS has proctoring and consultancy agreements with phenox and Balt international. The remaining authors declare that the research was conducted in the absence of any commercial or financial relationships that could be construed as a potential conflict of interest.
